# Reversible and Noisy Progression towards a Commitment Point Enables Adaptable and Reliable Cellular Decision-Making

**DOI:** 10.1371/journal.pcbi.1002273

**Published:** 2011-11-10

**Authors:** Anna Kuchina, Lorena Espinar, Jordi Garcia-Ojalvo, Gürol M. Süel

**Affiliations:** 1 Green Center for Systems Biology, University of Texas Southwestern Medical Center, Dallas, Texas, United States of America; 2 Department of Pharmacology, University of Texas Southwestern Medical Center, Dallas, Texas, United States of America; 3 Departament de Física i Enginyeria Nuclear, Universitat Politècnica de Catalunya, Terrassa, Spain; Stanford University, United States of America

## Abstract

Cells must make reliable decisions under fluctuating extracellular conditions, but also be flexible enough to adapt to such changes. How cells reconcile these seemingly contradictory requirements through the dynamics of cellular decision-making is poorly understood. To study this issue we quantitatively measured gene expression and protein localization in single cells of the model organism *Bacillus subtilis* during the progression to spore formation. We found that sporulation proceeded through noisy and reversible steps towards an irreversible, all-or-none commitment point. Specifically, we observed cell-autonomous and spontaneous bursts of gene expression and transient protein localization events during sporulation. Based on these measurements we developed mathematical population models to investigate how the degree of reversibility affects cellular decision-making. In particular, we evaluated the effect of reversibility on the 1) reliability in the progression to sporulation, and 2) adaptability under changing extracellular stress conditions. Results show that reversible progression allows cells to remain responsive to long-term environmental fluctuations. In contrast, the irreversible commitment point supports reliable execution of cell fate choice that is robust against short-term reductions in stress. This combination of opposite dynamic behaviors (reversible and irreversible) thus maximizes both adaptable and reliable decision-making over a broad range of changes in environmental conditions. These results suggest that decision-making systems might employ a general hybrid strategy to cope with unpredictably fluctuating environmental conditions.

## Author Summary

Cells must continuously make decisions in response to changes in their environment. These decisions must be irreversible, to prevent cells from reverting back to unfit cellular states, but also be flexible, to allow cells to go back to their previous state upon environmental changes. Using single-cell time-lapse fluorescence microscopy, we show that these seemingly contradictory properties coexist in *Bacillus subtilis* cells during their progression to spore formation. We suggest, on the basis of a mathematical population model, that reversible progression towards the irreversible decision to sporulate optimizes respectively adaptability and reliability of decision-making over a broad range of changes in environmental conditions.

## Introduction

Cellular decision-making underlies many biological processes such as multipotent differentiation, where cells commit to one of several distinct fates. Such cell fate choice must permit individual cells to reach a decision even in fluctuating extracellular environments [1]. At the same time, cells must also be able to adapt their cell fate choice to changes in these conditions. It is unclear how individual cells reconcile these opposing requirements of decisiveness and adaptability during decision-making. Decisive cellular differentiation mechanisms have been proposed to combine ultra-sensitivity and positive feedback to generate an irreversible and all-or-none cell fate choice such as those observed during *Xenopus* oocyte maturation [2] and yeast mating decision [3]. However, individual cells with irreversible responses can lack the flexibility to respond proportionally to changing environments, since even small changes can trigger irreversible responses. In contrast, progression of cellular differentiation through reversible intermediate states permits flexibility and proportional responses to environmental changes. For example, multipotent differentiation of hematopoietic stem cells is a stepwise process with numerous reversible intermediate states that allows cells to gradually adapt to changes in extracellular signals [4], [5], [6], [7], [8], [9], [10]. Despite these recent insights, how multipotent differentiation systems reach a decisive cell fate choice while maintaining the ability to respond to changes in the environment is largely unknown.

To understand cellular decision-making it is critical to determine the single-cell dynamics underlying the progression to cell fate choice. However, these dynamics are poorly characterized in multipotent differentiation systems ranging from bacteria to mammalian stem cells. Simultaneous measurement of multiple components of a differentiation program in the same cell can reveal the dynamics of cellular decision-making underlying multipotent differentiation. The soil bacterium *Bacillus subtilis* serves as an ideal model system in which the dynamics of multiple genes within a differentiation circuit are simultaneously measurable in single cells [11], [12], [13]. In stressful environments the majority of *B. subtilis* cells form spores that survive environmental extremes [14], [15]. The sporulation program has been well characterized genetically and multiple stages of sporulation have been described [16], [17], [18], [19], [20]. However, despite these important insights, how individual cells proceed to spore formation and thus the dynamics of the sporulation program in single cells has not been determined.

To uncover cell fate choice dynamics in *B. subtilis*, we simultaneously measured the activities of multiple sporulation circuit elements with single-cell resolution. We found that individual cells progress gradually to spore formation through reversible activities of early sporulation components. These measurements also allowed us to confirm the previously identified commitment point of cellular decision-making at which cells irreversibly proceed to complete spore formation [21]. More importantly, single cells analysis revealed the precise all-or-none dynamics of this decision-making point that was obscured by variability at the single-cell level in population measurements. Modeling of alternative sporulation dynamics showed that the combination of reversible and irreversible dynamics employed by *B. subtilis* can represent a general strategy to maximize reliable and yet adaptable cellular decision-making over a broad range of randomly fluctuating environmental conditions.

## Results

### Single cells of *Bacillus subtilis* progress reversibly and gradually to spore formation

**Figure 1:**
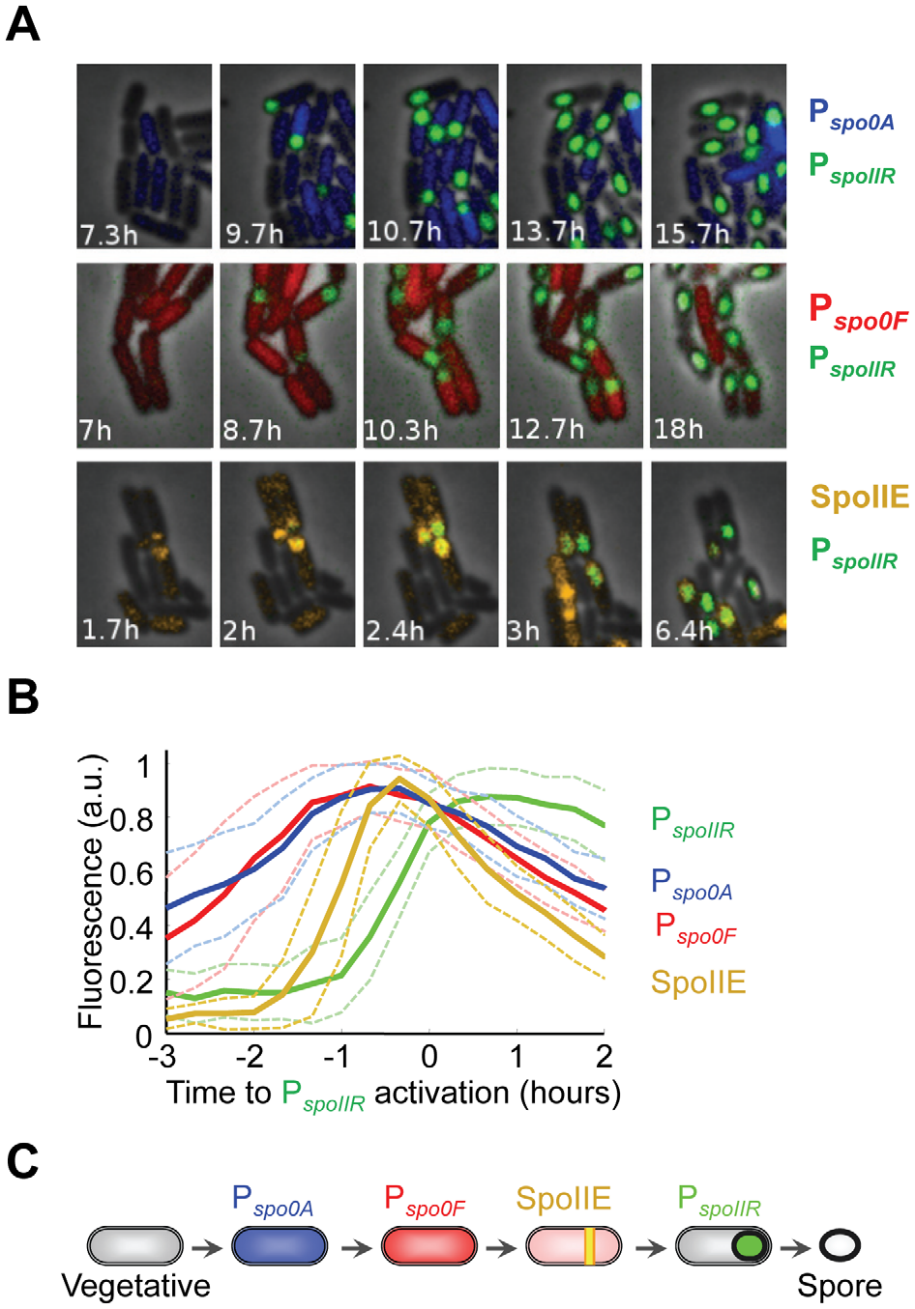
Reversibility of sporulation component activities during the progression to spore formation. *(A)* Filmstrips of phase and fluorescence image overlays of sporulating *B. subtilis* cells expressing pair-wise combinations of fluorescent proteins. For all panels, CFP fluorescence expressed from the P_spoIIR_ promoter is colored in green. In the same cells, YFP is expressed from the P*_spo0A_* promoter (upper panel, blue), from the P*_spo0F_* promoter (middle panel, red), and as a translational fusion to SpoIIE protein (lower panel, orange). Time is indicated in hours. *(B)* Mean quantitative time traces of sporulation reporters during typical sporulation events. Dynamics of the reporters were obtained from strains expressing pair-wise combinations of indicated sporulation reporters (strains 0A-IIR, n = 33; 0F-IIR, n = 30; IIE-IIR, n = 28; strain definitions and genetic background can be found in Supporting Text S2 and Supporting Table S1) and aligned in time with respect to activation of the common P*_spoIIR_* reporter (defined by P*_spoIIR_* fluorescence higher than 70% of maximum P*_spoIIR_* fluorescence at sporulation). Activities of all reporters were measured as mean fluorescence intensities in single cells. All traces have been normalized for amplitude. Dashed lines above and below the mean curves indicate standard deviation (SD). See also Supporting Fig. S1. *(C)* Scheme representing the temporal sequence of cellular states during the progression to sporulation, summarizing data from the plot shown in (*B*).

First, we established the single-cell dynamics of the progression toward spore formation by measuring the temporal profile of four sporulation components (Fig. 1*A*). Initiation of sporulation is controlled by a multicomponent phosphorelay including two phosphotransferases, Spo0F and Spo0B, and a transcription factor Spo0A [14], [19], [22], [23], [24], [25]. Spo0F protein senses and integrates a variety of inputs and as a result becomes phosphorylated [23], [24]. The phosphate group is subsequently transferred through Spo0B to Spo0A, a master regulator of sporulation, which upon phosphorylation directly controls expression of over 120 genes [20], [26], [27], including *spo0F* (in what constitutes a feedback mechanism [28], [29], [30]). Among these is the gene for SpoIIE, a protein phosphatase that is required for the activation of the forespore specific transcription factor (σ^F^) and whose localization to the asymmetric septum is the first morphological marker for forespore formation [31], [32], [33], [34], [35]. Activation of σ^F^ in the forespore switches on the expression of SpoIIR, which in turn leads to the activation of σ^E^, a transcription factor specific to the mother cell that switches on the expression of a large number of late sporulation genes [36], [37], [38]. At this point, the cell becomes irreversibly committed to sporulation [21], [37], [38], [39]. Using fluorescent reporter constructs, we quantified the activities of the Spo0A (P*_spo0A_*), Spo0F (P*_spo0F_*) and SpoIIR (P*_spoIIR_*) promoters (Fig. 1*A*, top two rows). We also visualized SpoIIE protein localization by utilizing a functional translational fusion to SpoIIE (Fig. 1*A*, bottom row). Each sporulation reporter was simultaneously measured in combination with the P*_spoIIR_* reporter. Measurement of these overlapping pair-wise combinations of reporters allowed us to establish a relative temporal profile for multiple sporulation steps with single-cell resolution (Fig. 1*B* and Supporting Fig. S1) that was consistent with the genetically established hierarchy within the sporulation circuit [14], [18], [22], [40], [41], [42] (Fig. 1*C*).

**Figure 2:**
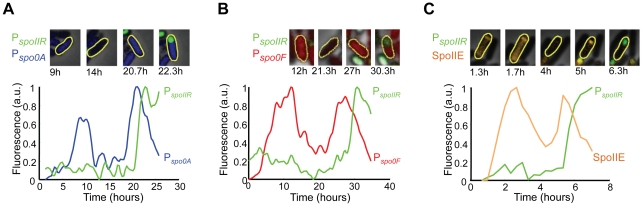
Smoothed quantitative time traces of normalized fluorescence signals reporting sporulation component activities in single cells. *(A)* 0A(5x)-IIR strain with P*_spo0A_*-CFP (blue) and P*_spoIIR_*-YFP (green). *(B)* 0F-IIR strain with P*_spo0F_*-YFP (red) and P*_spoIIR_*-CFP (green). *(C)* IIE-IIR strain showing localization of fusion protein SpoIIE-YFP (orange) and P*_spoIIR_*-CFP (green). For SpoIIE-YFP, local fluorescence measurements near the cell poles are shown. On top of each panel, cell images are shown at sample time points. The measured cell is indicated with a yellow outline.

Single cell measurements of sporulation reporters revealed that progression to spore formation is comprised of reversible steps. During the early progression to spore formation, individual cells exhibited bursts of P*_spo0A_* gene expression that did not result in spore formation (Fig. 2*A*). Activation of Spo0A during *B. subtilis* sporulation is known to be heterogeneous among single cells [43], [44]. However, spontaneous bursts of gene expression at single-cell level during sporulation have not been described to date. Bursts in gene expression were not limited to P*_spo0A_*, but were also observed for P*_spo0F_* (Fig. 2*B*). Given that P*_spo0F_* is, as mentioned above, transcriptionally activated by phosphorylated Spo0A, the dynamics of that promoter is reporting the activity of Spo0A. Consequently, the observed bursts in P*_spo0F_* indicate that not only the expression, but also the activity of the Spo0A master regulator exhibits bursts in single cells. In contrast, bursts of gene expression were not observed for the late stage sporulation reporter P*_spoIIR_* (Fig. 2*A–C*): after the sharp signal increase observed for P*_spoIIR_*, a spore is always formed (and thus the time traces shown in the plots cannot be continued). Gene expression bursts can introduce variability and reversibility during the early stages of the progression to spore formation, whereas the later stages do not appear to be subject to such stochasticity. Additionally, we observed reversible protein localization of SpoIIE in approximately 2±1% (SEM) of cells (Fig. 2*C*). Specifically, we observed transient localization events of the SpoIIE-YFP fusion protein to the asymmetric septum that did not give rise to spore formation. In these cells, SpoIIE either switched its localization between opposite poles, or completely delocalized and cells continued with cell division (Fig. 2*C*). Similar bursts of promoter activity and protein localization in single cells have also been reported in other systems, and have been attributed to the stochastic and reversible nature of the underlying biochemical reactions [43], [45], [46], [47], [48], [49], [50].

The single-cell reversibility observed here is distinct from what has been reported in population-level studies which showed that sporulation can be aborted upon transfer from stress to rich media conditions [21]. In contrast, the single-cell reversibility discussed here appears to be a cell autonomous behavior that occurs randomly without requiring a change in environmental conditions. Such cell autonomous and random sampling of heterogeneous behavior in single cells has also been observed in other systems where it may provide a fitness advantage, such as the generation of antibiotic resistant *Escherichia coli* persister cells [51] and the survival of *Saccharomyces cerevisiae* to cellular stress [50]. Together, our data reveal that the early steps of progression towards spore formation are subject to stochastic reversibility at the single-cell level, suggesting an adaptable progression to spore formation.

### *Bacillus subtilis* commit to sporulation in an all-or-none fashion

**Figure 3:**
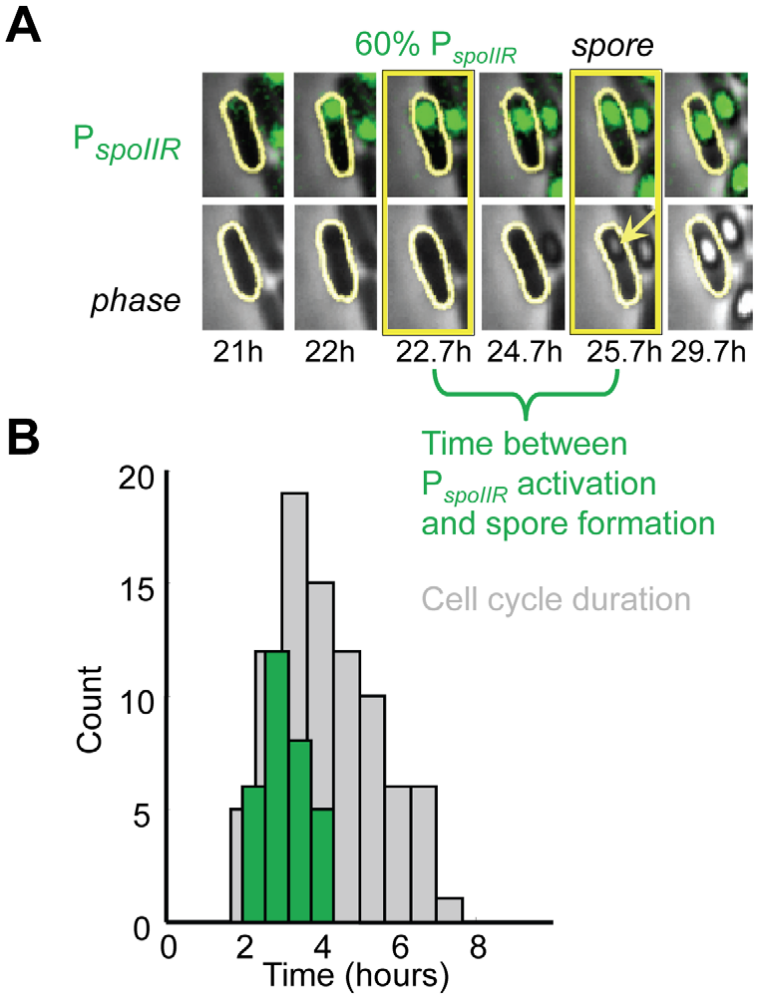
Precise timing of P*_spoIIR_* activation prior to spore formation. *(A)* Filmstrips showing fluorescence (top) and phase (bottom) images of a sample sporulating cell (strain 0A-IIR) activating P*_spoIIR_* expression in the forespore (green). Phase bright spore appearance is indicated by an arrow. *(B)* Histogram presenting, in green, the distribution of time between P*_spoIIR_* activation and phase bright spore formation (n = 31, sample events highlighted by yellow boxes in the filmstrips in panel (A)), compared to sample cell cycle durations, in light grey, measured in the same cells under movie-making conditions. P*_spoIIR_* activation is measured as >60% of maximum P*_spoIIR_* fluorescence at sporulation.

Detailed analysis of single cell dynamics revealed that the irreversible commitment for spore formation is executed within a narrow time window. Specifically, we find that P*_spoIIR_* activation (which as mentioned above, denotes the sporulation commitment point) is switch-like (Fig. 2*A–C*, green line). This timing was quantified by using the morphological appearance of an actual spore (indicated by a phase bright spot in our single-cell movies) as a reference time point from which to measure commitment time (Fig. 3*A*). Analysis of single-cell data shows that the temporal distance between P*_spoIIR_* activation and the formation of the morphologically visible forespore is tightly distributed (CV = 0.2, and 15% of median cell cycle duration) (Fig. 3*B*). These results show the precise timing of the irreversible decision and the completion of the spore formation process. Therefore, single cell measurements of sporulation dynamics exposed a precise cell intrinsic decision point in time that revealed switch-like dynamics of the commitment to spore formation. The temporal precision of this decision-making point was previously concealed in population measurements (see Fig. 1*B*) by the cell-to-cell variability in the reversible progression to spore formation.

### Mathematical modeling reveals advantage of *B. subtilis* cellular decision-making dynamics under alternating stress conditions

**Figure 4:**
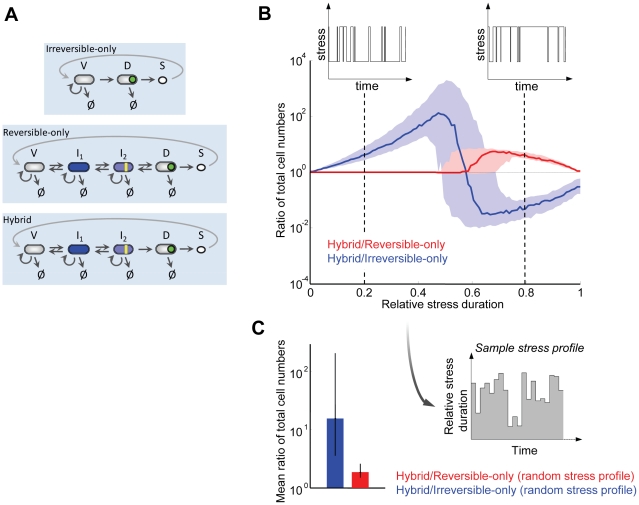
Mathematical comparison of distinct cell-fate choice dynamics under alternating environmental conditions. *(A)* Schematic representation of the three models.*Irreversible-only*, top panel: Decision to sporulate is made by cells in a single step. The model comprises three cellular states: vegetative (V), decided (D) and spores (S). *Reversible-only*, middle panel: In this model vegetative cells (V) proceed toward sporulation through two intermediate states I_1_ and I_2_. All transitions, including the transition to the decided state (D), are reversible. *Hybrid*, lower panel: This model combines characteristics from the two mechanisms described above. In this scenario, cells initially progress toward sporulation through intermediate states I_1_ and I_2_ with reversible transitions, with the irreversible final transition to the decided state (D). For all models the transition rates between sporulation stages, as well as growth and death (Ø) rates, are coupled to the stress level. For details, see Supporting Text S1. *(B)* Relative fitness of the hybrid model with respect to the irreversible-only and reversible-only models described in Fig. 3*A*. The blue and red lines correspond to the ratio of the total cell populations (summed over all cell states) of the hybrid model and the irreversible-only and reversible-only models, respectively. A random dichotomic variation of the environment is applied to all models, with the relative average duration of high versus low stress phases increasing in the x axis. The two insets depict sample profiles of stress for relative average durations of high stress phase equal to 0.2 and 0.8. The shaded areas in light blue and light red represent the maximum variation resulting from simultaneous random changes in all non-zero parameters of up to 20% around their baseline values given in Supporting Table S2. *(C)* Average fitness of the hybrid model with respect to the irreversible-only and reversible-only models obtained from a random sampling of environmental conditions. The inset shows short sample series of the average stress levels used to study the response of the three models to random stress conditions. Relative stress durations where chosen from a uniform random distribution in the range [0, 1]. The blue and red bars represent the ratio of the total cell population of the hybrid model to that of irreversible-only and reversible-only, respectively, averaged over 100 values of the relative average duration of high versus low stress scanned in (*B*). For each stress level, the models were integrated over 30 hours, in the same conditions as in (*B*). The error bars represent the maximum variation resulting from parameter changes up to 20% their baseline values, as in (*B*).

Our measurements suggest that the decision to sporulate in *B. subtilis* is made in an irreversible all-or-none manner, following a reversible and gradual progression toward this decision point. Therefore, sporulation combines reversible and irreversible dynamics, two seemingly opposed decision mechanisms. In order to examine the potential advantages of this hybrid mechanism, we compared the response of three simplified models of cellular decision-making to a variable-stress environment. These models describe the dynamics of a population of cells progressing toward sporulation under stress, in terms of the number of cells existing at any given time in a given state along the progression, beginning with the vegetative state and ending in the spore state. The dynamics of cell populations in all these states is given by a set of coupled ordinary differential equations that are linear, and thus can be solved exactly (Supporting Text S1). The three models, shown schematically in Fig. 4*A* and described in detail in the Supporting Information (Supporting Text S1 and Supporting Fig. S2), involve either 1) a purely irreversible/all-or-none, 2) a purely reversible/gradual, or 3) a “hybrid” process that takes cells from their initial vegetative state to their final spore state. In the irreversible-only scheme, cells decide in a single step whether or not to sporulate, and the decision is irreversible. The reversible-only model takes cells gradually toward sporulation through multiple reversible intermediate states, without any irreversible commitment step taking place along the process. Only the ultimate transition to the spore state, taking place *after* the decision, is irreversible. Finally, the third “hybrid” model features the actual sporulation dynamics identified here for *B. subtilis* that combines a gradual progression through reversible intermediate states with an irreversible all-or-none decision prior to the spore transition.

We subjected the three models described above to a random variation of the environment, with alternation of a high and a low level of stress (insets in Fig. 4*B* and Supporting Fig. S3). For all models, the rates of progression toward/back from sporulation, growth and death rates are all coupled to the level of stress (Supporting Text S1). We then systematically varied the ratio of high stress to total cycle duration, which was kept constant. The relative fitness of the hybrid model with respect to the irreversible-only and reversible-only models was measured as the ratio of total cells in the population between the models (Fig. 4*B*, blue and red lines respectively).

Results of this analysis show that the survival rate of the different models depends on the stress profile. None of the models dominates over the entire range of environmental stress conditions, but the hybrid model performs best overall. For short phases of high stress, the hybrid outgrows the irreversible-only model (left half of main plot in Fig. 4*B*, blue line), since the latter is driven irreversibly to sporulation even for short stress periods. Under these conditions, the hybrid and reversible-only models perform similarly (red line) since in both models reversible progression delays sporulation. However, in the opposite limit where high stress durations approach the total duration (right half of main plot in Fig. 4*B*), the irreversible-only model outgrows the hybrid model, since responsiveness is a disadvantage. For such prolonged high stress durations, spores are at an advantage because non-spore cells have a higher death than growth rate under stress. In this limit, the hybrid model is in turn more reliable than the reversible-only model, since the reversible-only model is driven away from sporulation even by short intervals of rich phase. Parameter sensitivity analysis showed that these results hold over a range of parameter values (Fig. 4*B*, light blue and light red regions). Taken together, these results show that the hybrid model outperforms both the irreversible-only and reversible-only schemes over a broader range of randomly alternating stress profiles, given its responsiveness to the long-term recovery from stress and its reliability during short-term release of stress conditions.

This prediction was tested directly by subjecting all three models to random changes in the fraction of time spent in high stress. In these extended simulations cells were exposed to 100 random samplings of environmental stress ratios (part of an environmental profile is shown in Fig. 4*C*, inset). We then calculated the ratio of the total cell population of the hybrid model to that of the irreversible-only and reversible-only models averaged over the entire simulation, defining the relative fitness of each model (Fig. 4*C*). Our data demonstrate that the hybrid model has an overall higher fitness over both the irreversible-only and reversible-only models when populations are subjected to a randomly changing range of environmental conditions. Therefore, the combination of gradual progression toward an all-or-none decision in *B. subtilis*, as represented in the hybrid model and observed experimentally, enables the system to cope with a broader range of unpredictable stress conditions.

## Discussion

Single cell measurements of sporulation components allowed us to precisely determine both the temporal progression of the earliest sporulation events and the consecutive switch-like dynamics of the commitment point. The temporal precision and switch-like nature of the decision point would have been concealed in population measurements that describe average behaviors of cells and cannot account for cell-to-cell variability. Similarly, a recent study of the post-infection decision in bacteriophage lambda to undergo lysis or lysogeny showed how cell-cell variability obscured the precision of cell fate choice [52]. Therefore, stochastic fluctuations and noise observed at the single-cell level can conceal the temporal precision of numerous cellular processes. Our approach based on measuring multiple gene circuit components simultaneously in the same cell can identify cell intrinsic temporal reference points that can accurately establish the relative timing of cellular processes.

Multipotent differentiation in *B. subtilis* appears to combine the opposing dynamic regimes of reversible/gradual and irreversible/all-or-none behavior to reconcile the seemingly contradictory requirements of adaptable and reliable cellular decision-making. The time required for cells to reach spore formation is highly variable, allowing cells to generate a broad distribution of wait times prior to cell fate choice. This extends the period during which *B. subtilis* cells are known to be responsive to environmental changes. Therefore, variability in sporulation progression can be beneficial biologically. These findings thus extend previous results regarding the advantageous role of stochasticity in enhancing survival under uncertain environments [13], [50], [53], [54], [55], [56]. In contrast, the actual decision to sporulate is governed by irreversible and all-or-none dynamics, which provides reliable execution of the sporulation program. Our modeling suggests that this combination of gradual and all-or-none dynamics during cell fate choice allows *B. subtilis* to successfully survive under a broad range of alternating environmental stress profiles. In mammalian cells, the decision for apoptosis has been described as a slow progression towards a fast decision, suggesting that a hybrid strategy similar to that described here might be employed [57]. Therefore, this strategy of combining opposing dynamic behaviors may be common to various other biological processes, and may represent a general mechanism for decision-making under unpredictably changing environments. Models for decision-making under uncertain conditions have been developed and applied to numerous unrelated complex systems, such as finance [58]. Perhaps a stochastically reversible progression to an irreversible all-or-none switch as observed here in cells, may also serve as a strategy for adaptable and reliable decision-making in other complex systems that are subject to unpredictable conditions.

## Materials and Methods

### Strain construction

*Bacillus subtilis* strains used in the study are isogenic to wild-type *B. subtilis* PY79 strain and are listed in Supporting Table S1. Promoter – fluorescent proteins fusions were generated using fusion polymerase chain reaction and cloned into *B. subtilis* chromosomal integration vectors following standard protocols. The following *B. subtilis* chromosomal integration vectors were used: pSac-Cm, integrating into the *sacA* locus (constructed by R. Middleton and obtained from the Bacillus Genetic Stock Center) and pLD30 integrating into the *amyE* locus (kind gift from Jonathan Dworkin, Columbia University). We have also utilized the bifunctional cloning plasmid pHP13 carrying the replication origin of the cryptic *B. Subtilis* plasmid pTA1060 (5 copies per genome) [59]. Standard one-step transformation protocol was followed to transform *B. subtilis* with these constructs.

### Growth and imaging conditions

For imaging, *B. subtilis* cells were grown at 37°C in LB with the following concentrations of appropriate antibiotic: 5 µg/ml chloramphenicol, 5 µg/ml neomycin, 5 µg/ml erythromycin or 100 µg/ml spectinomycin. After reaching OD 1.8, the cells were resuspended in 0.5 volume of Resuspension Media (RM; see Supporting Text S2, Materials and Methods) supplemented with 0.02% glucose. The cells were incubated at 37°C for 1 hour, then diluted 10-fold in RM and applied onto a 1.5% low-melting agarose pad placed into a coverslip-bottom Willco dish for imaging.

### Time-lapse microscopy

Growth of microcolonies was observed with fluorescence time-lapse microscopy at 37°C with an Olympus IX-81 inverted microscope with a motorized stage (ASI) and an incubation chamber. Image sets were acquired every 20 min with a Hamamatsu ORCA-ER camera. The imaging time has been optimized in order to prevent phototoxicity [11]. Custom Visual Basic software in combination with the Image Pro Plus (Media Cybernetics) was used to automate image acquisition and microscope control.

### Image analysis

Time-lapse movie data analysis was performed by custom software developed with MATLAB image processing and statistics toolboxes (The Mathworks) described in [60] and [11].

### Mathematical modeling

Extended description of the models and methods is available in Supporting Text S1, Mathematical Modeling.

## Supporting Information

Figure S1 Single-cell traces of sporulating cells expressing pair-wise combinations of fluorescent sporulation markers. In panels (*A*) through (*C*), quantitative time traces of sporulation reporters during typical sporulation events are shown in lighter colors, with mean trace shown on top in bright color and dashed lines indicating standard deviation (SD). Dynamics of the reporters in each panel were obtained from strains expressing pair-wise combinations of indicated sporulation reporters: (*A*) strain 0A-IIR, n = 33, (*B*) strain 0F-IIR, n = 30, (C) strain IIE-IIR, n = 28. All traces were aligned with respect to P*_spoIIR_* activation (in green) defined as >70% of fluorescence intensity relative to maximum intensity at sporulation. Data from these panels were combined to produce Fig. 1*B*.
(PDF)

Figure S2 Three alternative models of sporulation progression.
(PDF)

Figure S3 Stress modulation.
(PDF)

Table S1 Strain definitions and genetic background. In the first column, promoters expressing fluorescent proteins are abbreviated as follows: “0A”, P*_spo0A_*; “0F”, P*_spo0F_*; IIE, spoIIE; “IIR”, P*_spoIIR_*. (5x) denotes average copy number of plasmid pHP13 (this strain was used where indicated in place of 0A-IIR for increased signal-to-noise ratio).
(PDF)

Table S2 Parameter values in the rich and poor phases.
(PDF)

Text S1 Mathematical modeling.
(PDF)

Text S2 Materials and methods.
(PDF)
