# Supplementary material for: Reversible and Noisy Progression towards a Commitment Point Enables Adaptable and Reliable Cellular Decision-Making
Source: PLoS Comput Biol. 2011 Nov 10;7(11):e1002273. doi: 10.1371/journal.pcbi.1002273 (PMC3213189; doi:10.1371/journal.pcbi.1002273)
Supplement: Figure S1 — Single-cell traces of sporulating cells expressing pair-wise combinations of fluorescent sporulation markers. In panels (A) through (C), quantitative time traces of sporulation reporters during typical sporulation events are shown in lighter colors, with mean trace shown on top in bright color and dashed lines indicating standard deviation (SD). Dynamics of the reporters in each panel were obtained from strains expressing pair-wise combinations of indicated sporulation reporters: (A) strain 0A-IIR, n = 33, (B) strain 0F-IIR, n = 30, (C) strain IIE-IIR, n = 28. All traces were aligned with respect to PspoIIR activation (in green) defined as >70% of fluorescence intensity relative to maximum intensity at sporulation. Data from these panels were combined to produce Fig. 1B . (PDF) [file pcbi.1002273.s001.pdf]

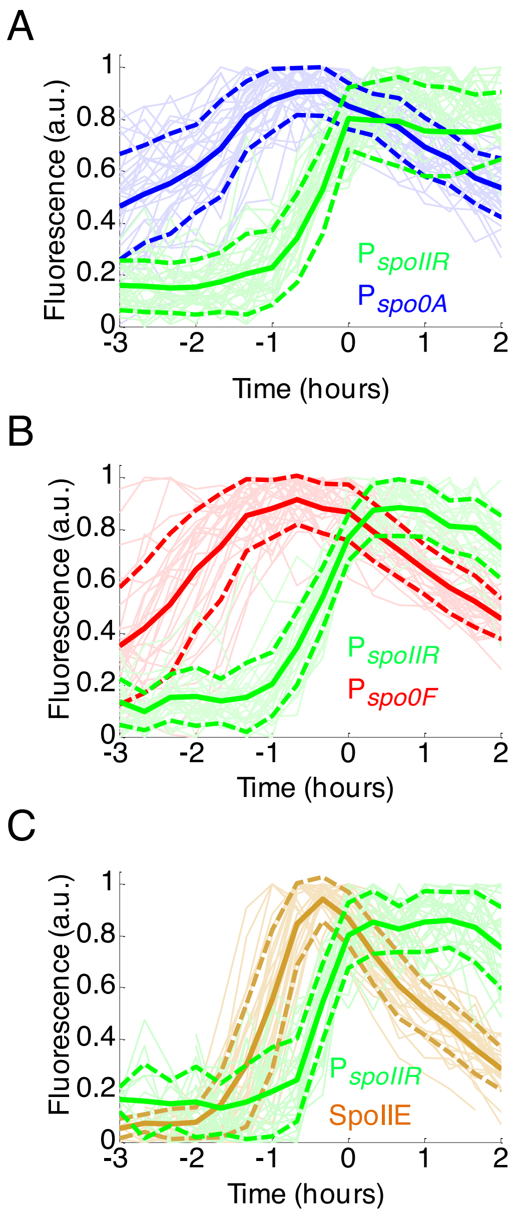

Figure S1. Single-cell traces of sporulating cells expressing pair-wise combinations of fluorescent sporulation markers. In panels (*A*) through (*C*), quantitative time traces of sporulation reporters during typical sporulation events are shown in lighter colors, with mean trace shown on top in bright color and dashed lines indicating standard deviation (SD). Dynamics of the reporters in each panel were obtained from strains expressing pair-wise combinations of indicated sporulation reporters: (*A*) strain 0A-IIR, n = 33, (*B*) strain 0F-IIR, n = 30, (C) strain IIE-IIR, n = 28. All traces were aligned with respect to P_{spoIIR} activation (in green) defined as >70% of fluorescence intensity relative to maximum intensity at sporulation. Data from these panels were combined to produce Fig. 1*B*.
