# Supplementary figures and images for: Reversible and Noisy Progression towards a Commitment Point Enables Adaptable and Reliable Cellular Decision-Making
Source: PLoS Comput Biol. 2011 Nov 10;7(11):e1002273. doi: 10.1371/journal.pcbi.1002273 (PMC3213189; doi:10.1371/journal.pcbi.1002273)

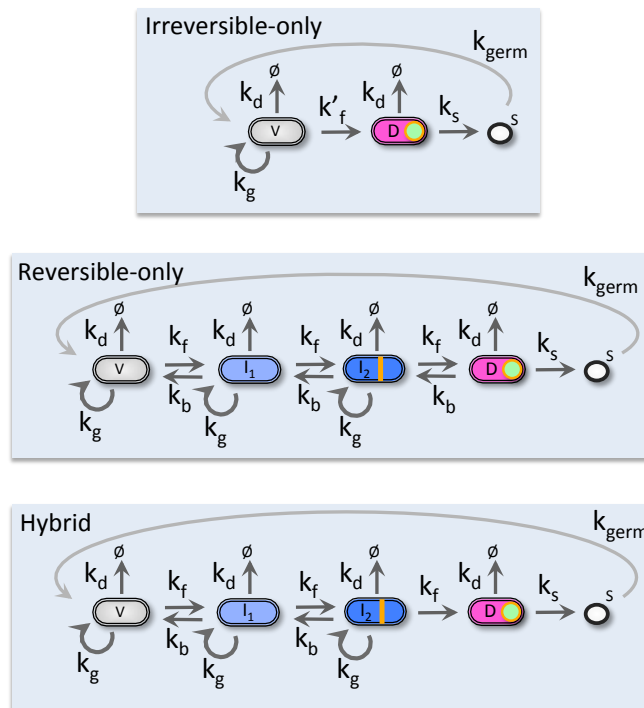

**Figure S2 | Three alternative models of sporulation progression.**

Supplement: Figure S2 — Three alternative models of sporulation progression. (PDF) [file pcbi.1002273.s002.pdf]

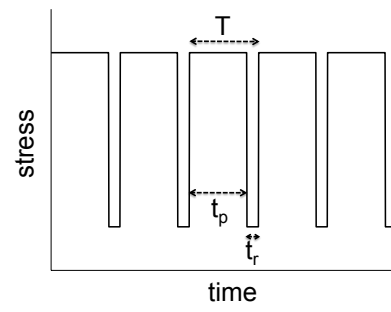

**Figure S3 | Stress modulation.**

Supplement: Figure S3 — Stress modulation. (PDF) [file pcbi.1002273.s003.pdf]
