# Supplementary material for: Reversible and Noisy Progression towards a Commitment Point Enables Adaptable and Reliable Cellular Decision-Making
Source: PLoS Comput Biol. 2011 Nov 10;7(11):e1002273. doi: 10.1371/journal.pcbi.1002273 (PMC3213189; doi:10.1371/journal.pcbi.1002273)
Supplement: Table S1 — Strain definitions and genetic background. In the first column, promoters expressing fluorescent proteins are abbreviated as follows: “0A”, Pspo0A; “0F”, Pspo0F; IIE, spoIIE; “IIR”, PspoIIR. (5x) denotes average copy number of plasmid pHP13 (this strain was used where indicated in place of 0A-IIR for increased signal-to-noise ratio). (PDF) [file pcbi.1002273.s004.pdf]

B. subtilis strains isogenic to P*Y79*	Genotype	Appearance in figures
0A-IIR	*AmyE*::P_{spo0A}-*yfp,* P_{comG}-*mCherry* (Sp^R)<br>*SacA*::P_{spoIIR}-*cfp* (Cm^R)	1*B,* 3, S3*A*
0F-IIR	*AmyE*::P_{spo0F}-*yfp* (Sp^R)<br>*SacA*::P_{spoIIR}-*cfp* (Cm^R)	1*A-B*, 2*B,* S3*B*
IIE-IIR	*AmyE*::P_{spoIIE}-*spoIIE-yfp* (Sp^R)<br>*SacA*::P_{spoIIR}-*cfp* (Cm^R)<br>ΔSpoIIE::Neo	1*A-B*, 2*C*, S3*C*
0A (5x)-IIR	*SacA*::P_{spoIIR}-*yfp* (Cm^R)<br>pHP13-P_{spo0A}-cfp (Erm^R)	1*A*, 2*A*

Table S1. Strain definitions and genetic background. In the first column, promoters expressing fluorescent proteins are abbreviated as follows: “0A”, P_{spo0A}; “0F”, P_{spo0F}; IIE, spoIIE; “IIR”, P_{spoIIR}. (5x) denotes average copy number of plasmid pHP13 (this strain was used where indicated in place of 0A-IIR for increased signal-to-noise ratio).
