# Supplementary material for: Reversible and Noisy Progression towards a Commitment Point Enables Adaptable and Reliable Cellular Decision-Making
Source: PLoS Comput Biol. 2011 Nov 10;7(11):e1002273. doi: 10.1371/journal.pcbi.1002273 (PMC3213189; doi:10.1371/journal.pcbi.1002273)
Supplement: Table S2 — Parameter values in the rich and poor phases. (PDF) [file pcbi.1002273.s005.pdf]

rate	value (rich phase)	value (poor phase)
k_f	0	0.6
k'_f	0	0.2
k_b	20	0
k_g	1	0.1
k_d	0.1	1
k_s	0	0.2
k_{germ}	0.1	0

Table S2 | Parameter values in the rich and poor phases.
