# Supplementary material for: Reversible and Noisy Progression towards a Commitment Point Enables Adaptable and Reliable Cellular Decision-Making
Source: PLoS Comput Biol. 2011 Nov 10;7(11):e1002273. doi: 10.1371/journal.pcbi.1002273 (PMC3213189; doi:10.1371/journal.pcbi.1002273)
Supplement: Text S1 — Mathematical modeling. (PDF) [file pcbi.1002273.s006.pdf]

Supporting Text S1

Reversible and noisy progression towards a commitment point enables adaptable and reliable cellular decision-making

Anna Kuchina * [1,2], Lorena Espinar * [3], Jordi Garcia-Ojalvo[3] and Gürol M. Süel[1,2]

** These authors contributed equally to this work*

[1]Green Center for Systems Biology and [2]Department of Pharmacology, University of Texas Southwestern Medical Center, Dallas, TX 75390

[3]Departament de Física i Enginyeria Nuclear, Universitat Politècnica de Catalunya, Edif. GAIA, Rambla Sant Nebridi s/n, E-08222 Terrassa, Spain

S1. MATHEMATICAL MODELING

S1.1 Population models of sporulation progression

In order to examine the potential advantages of reversible progression to cell fate choice, we compared the response of three simplified models of cellular decision-making to a variable-stress environment. These models describe the dynamics of a population of cells progressing toward sporulation under stress, in terms of the number of cells existing at any given time in a given state along the progression, beginning with the vegetative state and ending in the spore state. The dynamics of cell populations in all these states is given by a set of coupled ordinary differential equations that are linear, and thus can be solved exactly (see Sections S1.3 and S1.4 below). The three models, shown schematically in Supporting Fig. S2, involve either a purely irreversible/all-or-none, purely reversible/gradual, or a hybrid process that take cells from their initial vegetative state to their final spore state. In the irreversible-only scheme, cells decide in a single step whether or not to sporulate, and the decision is irreversible. The reversible-only model takes cells gradually toward sporulation through multiple reversible intermediate states, without any real decision taking place along the process. Only the ultimate transition to the spore state, taking place after the decision, is irreversible. Finally, the third model features the actual sporulation dynamics identified here for *B. subtilis* that combines a gradual progression through reversible intermediate states with an irreversible all-or-none decision prior to the spore transition.

S1.2 Model description

The population dynamics of the cell population as it progresses towards sporulation is given by the following linear dynamical equations:

$$\frac{d\mathbf{n}}{dt} = \mathrm{A}\mathbf{n} \qquad \text{(S1)}$$

where $\mathbf{n}$ is the state vector of the system giving the number of cells in the different states, and A is the matrix that contains the transition rates between cell populations (in what follows we represent vectors by boldface lowercase letters and matrices by uppercase letters). As described in Section S1.1 above, we consider three different schemes of sporulation progression, shown in Supporting Fig. S2. For each scheme, the structures of $\mathbf{n}$ and A are different, as we now describe.

- In the *irreversible-only* model, cells decide in a single step whether or not to sporulate. The model comprises three distinct cellular states: vegetative (V), decided (D), and spore (S). The corresponding state vector is thus three dimensional, $\mathbf{n} = (v, d, s)^T$

(with the superindex 'T' indicating transposition of the vector). The transition matrix has the form:Su

$$\mathsf{A} = \begin{pmatrix} -k'_f - k_d + k_g + k_{\text{germ}} & 0 & 0 \\ k'_f & -k_d - k_s & 0 \\ 0 & k_s & -k_{\text{germ}} \end{pmatrix}$$

Note that the rank of A is 2, because the spore dynamics does not affect the other two variables. The number of spores is merely a readout of the cell population in the presence of sustained stress. The transition rates on which A depends are defined in Supporting Fig. S2.

- In the *reversible-only model*, the transition from vegetative cells to spores occurs in a multi-step manner. Here we assume two distinct intermediate states (I_1 and I_2). Note that there is no all-or-none decision in this case, because the final transition to the decided state (D) is reversible. In this scenario, cells simply "leak" towards sporulation while they are in the so-called decided state. The state vector is now five-dimensional, $\mathbf{n} = (v, i_1, i_2, d, s)^T$. The transition matrix has the form:

$$\mathsf{A} = \begin{pmatrix} -k_f - k_d + k_g + k_{\text{germ}} & k_b & 0 & 0 & 0 \\ k_f & k_g - k_d - k_b - k_f & k_b & 0 & 0 \\ 0 & k_f & k_g - k_d - k_b - k_f & k_b & 0 \\ 0 & 0 & k_f & -k_s - k_d - k_b & 0 \\ 0 & 0 & 0 & k_s & -k_{\text{germ}} \end{pmatrix}$$

- The *hybrid model* combines characteristics of both the irreversible-only and reversible-only models described above. On the one hand, the model is irreversible because the transition towards the decided state is all-or-none. On the other other, the model progresses gradually towards that decision via intermediate steps. The state vector is again five-dimensional, $\mathbf{n} = (v, i_1, i_2, d, s)^T$, and the transition matrix now takes the form:

$$\mathsf{A} = \begin{pmatrix} -k_f - k_d + k_g + k_{\text{germ}} & k_b & 0 & 0 & 0 \\ k_f & k_g - k_d - k_b - k_f & k_b & 0 & 0 \\ 0 & k_f & k_g - k_d - k_b - k_f & 0 & 0 \\ 0 & 0 & k_f & -k_s - k_d & 0 \\ 0 & 0 & 0 & k_s & -k_{\text{germ}} \end{pmatrix}$$

S1.3 Temporal evolution in a constant environment

Since Eq. (S1) is linear, it can be solved in a straightforward way in the case of a constant environment (i.e. for constant reaction rates). The solution is simply

$$\mathbf{n}(t) = \sum_{i=1}^{d} c_i \mathbf{v_i} \exp(\lambda_i t)\,, \tag{S2}$$

where d denotes the dimension of the square matrix A, whose eigenvalues are represented by λ_i and the eigenvectors by $\mathbf{v_i}$. The scalars c_i are integration constants that depend on the initial condition of the trajectory. This solution can be expressed in a more compact form by defining the $d \times d$ matrix of eigenvectors V (whose columns are the eigenvectors of A), the $d \times d$ matrix of eigenvalues Λ (a diagonal matrix whose diagonal elements are the eigenvalues of A), and the d-dimensional vector $\mathbf{c}$ of integration constants. With those definitions, the solution (S2) can be written as

$$\mathbf{n}(t) = \mathsf{V} \exp\left(\Lambda t\right) \mathbf{c} \tag{S3}$$

The expression that gives the values of the integration constants in terms of the initial conditions is simply

$$\mathbf{n}(0) = \mathsf{V}\mathbf{c} \quad \Longrightarrow \quad \mathbf{c} = \mathsf{V}^{-1}\mathbf{n}(0)$$

Thus the final expression for the system's dynamics is simply:

$$\mathbf{n}(t) = \mathsf{V} \exp\left(\Lambda t\right) \mathsf{V}^{-1}\mathbf{n}(0) \tag{S4}$$

S1.4 Temporal evolution under dichotomic stress cycles

In order to examine the response of each of the three models described above to a varying environment, we now consider a random dichotomic variation of the stress as shown in the main text. In this scenario, the stress fluctuates randomly between two values, with the low-stress (rich) and high-stress (poor) phases lasting a time that follows an exponentially decreasing distribution corresponding to a random Poisson process.

The two phases have distinct values of the reaction parameters defined in Supporting Fig. S2. For instance, we assume that during the low-stress phase there is no progression towards sporulation ($k_f = k_s = 0$) and the death rate is very small in comparison with the growth rate. Oppositely, during the high-stress phase we consider that there is no possibility to reverse the sporulation progression ($k_b = 0$), while the growth rate is greatly diminished with respect

to the rich-medium condition. The specific parameter values used in this paper are given in Supporting Table S2. We note that k_f and k'_f are chosen such that the average time to the decision (from V to D) is the same in all models (i.e. $k'_f = k_f/3$).

Let T be the total duration of the environmental cycle, and t_p and t_r the durations of the poor and rich phases, respectively (see Supporting Fig. S3). After the poor phase of the first cycle, the population is, according to Eq. (S4),

$$\mathbf{n}(t_p) = \mathsf{V_p} \exp\left(\Lambda_\mathsf{p} t_p\right) \mathsf{V_p}^{-1} \mathbf{n}(0) \tag{S5}$$

This value is now used as initial condition of the following rich phase, after which the population is:

$$\mathbf{n}_1 = \mathsf{V_r} \exp\left(\Lambda_\mathsf{r} t_r\right) \mathsf{V_r}^{-1} \mathsf{V_p} \exp\left(\Lambda_\mathsf{p} t_p\right) \mathsf{V_p}^{-1} \mathbf{n}_0 \,, \tag{S6}$$

where the subindex in $\mathbf{n}$ denotes now the cycle. After q cycles, the population is thus

$$\mathbf{n}_q = \left(\mathsf{V_r} \exp\left(\Lambda_\mathsf{r} t_r\right) \mathsf{V_r}^{-1} \mathsf{V_p} \exp\left(\Lambda_\mathsf{p} t_p\right) \mathsf{V_p}^{-1}\right)^q \mathbf{n}_0 \,. \tag{S7}$$
