# Supplementary material for: Reversible and Noisy Progression towards a Commitment Point Enables Adaptable and Reliable Cellular Decision-Making
Source: PLoS Comput Biol. 2011 Nov 10;7(11):e1002273. doi: 10.1371/journal.pcbi.1002273 (PMC3213189; doi:10.1371/journal.pcbi.1002273)
Supplement: Text S2 — Materials and methods. (PDF) [file pcbi.1002273.s007.pdf]

# Supporting Text S2

# Reversible and noisy progression towards a commitment point enables adaptable and reliable cellular decision-making

Anna Kuchina * [1,2], Lorena Espinar * [3], Jordi Garcia-Ojalvo[3] and Gürol M. Süel[1,2]

** These authors contributed equally to this work*

*[1]Green Center for Systems Biology and [2]Department of Pharmacology, University of Texas Southwestern Medical Center, Dallas, TX 75390*

*[3]Departament de Física i Enginyeria Nuclear, Universitat Politècnica de Catalunya, Edif. GAIA, Rambla Sant Nebridi s/n, E-08222 Terrassa, Spain*

# S2. MATERIALS AND METHODS

### S2.1 Strain construction

*Bacillus subtilis* strains used in the study are isogenic to wild-type *B. subtilis* PY79 strain and are listed in Table S1. Polymerase Chain Reaction (PCR) was utilized to amplify native $P_{spo0A}$, $P_{spo0F}$, $P_{spoIIE}$-*spoIIE,* and $P_{spoIIR}$ from *B. subtilis* PY79 strain with the following primers:

| | |
|---|---|
| $P_{spo0A}$ | AATGTGACTTTCCTCCTTGTTTCTTCCTCCCCAAATGTAGTTAACAG and AGGAATTCGAAAAAGCAAGCTGACTGCCG |
| $P_{spo0F}$ | ATGGATCCCAGAAAGTCAAAAGCCTCCGAGCCGGAGGCTTTTGACTA TTACTCAAGTGAATCCTCCTTTATAACGTACAATATCAGTATAC (containing a terminator sequence) and CTTTGCTCATAATGTGACTTTC CTCCTTTTTACACCCCAATATTATGATTTTCGTCAAAAG |
| $P_{spoIIE}$-*spoIIE* | TTGGATCCGTACGGGTCATCCTAACAAATCGG and CGGTGAACAGTTCTT CACCTTTGCTTGAAATTTCTTGTTTGTTTTGAAAGATTG |
| $P_{spoIIR}$ | TCGGATCCTGATAACAGCACTGTATAATTGTATTTCATC and CGCATTATGTGACTTTCCTCCTTTCTAGCCCTATTATGGACAAGGAG |

Amplified fragments were cloned into *B. subtilis* chromosomal integration vectors: pSac-Cm, integrating into the *sacA* locus (constructed by R. Middleton and obtained from the Bacillus Genetic Stock Center) and pLD30 designed to integrate into the *amyE* locus (kind gift from Jonathan Dworkin, Columbia University); we have also used plasmid pHP13 carrying the replication origin of the cryptic *B. Subtilis* plasmid pTA1060 (5 copies per genome) (1). Standard *B. subtilis* transformation protocols were followed to transform *B. subtilis* strain PY79 with these constructs.

### S2.2 Imaging and growth conditions

#### S2.2.1 Preparation for microscopy

For imaging, *B. subtilis* cells were grown at $37^0$C in LB with appropriate selection. Antibiotics for selection were added to the following final concentrations: 5 μg/ml chloramphenicol, 5 μg/ml

neomycin, 5µg/ml erythromycin and 100 µg/ml spectinomycin. Cells grown to OD 1.8 were resuspended in 0.5 volume of Resuspension Media (RM; composition [per 1 liter]: 0.046 mg $FeCl_2$, 4.8 g $MgSO_4$, 12.6 mg $MnCl_2$, 535 mg $NH_4Cl$, 106 mg $Na_2SO_4$, 68 mg $KH_2PO_4$, 96.5 mg $NH_4NO_3$, 219 mg $CaCl_2$, 2 g L-glutamic acid)(2) supplemented with 0.02% glucose. The cells were incubated at $37^0$C for 1 hour, then diluted 10-fold in RM and applied onto a 1.5% low-melting agarose pad placed into a coverslip-bottom Willco dish for imaging. This protocol is optimized for time-lapse microscopy. RM reduces the growth rate of microcolonies on agarose and leads to sporulation. The imaging was performed as described previously (3).

### S2.2.2 Time-lapse microscopy and image analysis

Growth of microcolonies was observed with fluorescence time-lapse microscopy at $37^0$C with an Olympus IX-81 inverted microscope with a motorized stage (ASI) and an incubation chamber. Image sets were acquired every 20 min with a Hamamatsu ORCA-ER camera. The imaging time has been optimized in order to prevent phototoxicity (3). Custom Visual Basic software in combination with the Image Pro Plus (Media Cybernetics) was used to automate image acquisition and microscope control.

Time-lapse movie data analysis was performed by custom software developed with MATLAB image processing and statistics toolboxes (The Mathworks) (3).

## Supplementary References


1. Haima P, Bron S, & Venema G (1987) The effect of restriction on shotgun cloning and plasmid stability in Bacillus subtilis Marburg. *Mol Gen Genet* 209(2):335-342.
2. Sterlini JM & Mandelstam J (1969) Commitment to sporulation in Bacillus subtilis and its relationship to development of actinomycin resistance. *Biochem J* 113(1):29-37.
3. Suel GM, Garcia-Ojalvo J, Liberman LM, & Elowitz MB (2006) An excitable gene regulatory circuit induces transient cellular differentiation. *Nature* 440(7083):545-550.
